# Preparation of laser microporous porcine acellular dermal matrix and observation of wound transplantation

**DOI:** 10.1007/s10561-022-10023-7

**Published:** 2022-07-09

**Authors:** Weidong Xia, Cai Lin, Zhuolong Tu, Yuan Li, Guoliang Shen

**Affiliations:** 1 The Burn Plastic Surgery, The First Affiliated Hospital of Soochow University, Soochow University, Suzhou, 215006 China; 2 Department of Burn, The First Affiliated Hospital of Wenzhou Medical University, Nan Bai Xiang, Wenzhou, 325000 Zhejiang China

**Keywords:** Acellular dermal matrix, Laser micropore, Skin tissue engineering, Transplantation

## Abstract

To prepare a new type of porcine acellular dermis matrix (PADM) with the new laser microporous technique and verify its safety and feasibility. A novel porcine acellular dermis matrix (ADM) was prepared by using sequential combined decellularization of trypsin, neutral protease and SDS solution method and fully rinsed with ultrasonic wave. Specific laser microporous technology was used to prepare the laser micropore porcine acellular dermal matrix (LPADM). SD rats were chose as the animal models and autologous skin was transplanted by one-step method to observe and detect the graft activity, immunogenicity and vascularization degree of the novel PADM. A porcelain white, shiny, soft and elastic dermal matrix was prepared in this study, the results showed low DNA residue and low cytotoxicity. HE staining and SEM observation revealed that the PADM had neither residual cells nor cell fragments, while the collagen bundles were intact and orderly arranged. All the SD rats survived. No infection or skin allergy was found after surgery. None of the animals lost weight. Histological examination showed that the LPADM was fully vascularized with little tissue destruction in the experiment group. Immunohistochemical staining for CD31 showed ideal vascularization in the experiment group, and immunohistochemical staining for TNF-α showed there were no statistical significance of inflammatory reaction in both groups. This study demonstrated that the novel PADM prepared by sequential combined decellularization of trypsin, neutral protease and SDS solution method and new laser microporous technique was effective and safe in animal transplantation.

## Introduction

Skin is among the largest and fastest self-renewal organs in the human body (Marino et al. 2014; Chanson et al. 2018). It is the first defensive line for protecting the body against infection, mechanical forces, fluid imbalance, and thermal dysregulation (Chambers and Vukmanovic-Stejic 2020; Sorg et al. 2017). Due to lack of transplantable autologous skin and dermal substitute, the wound repair of acute and chronic injuries caused by inflammation, ulcers, severe burns, wounds, as well as other large areas of skin defects and refractory skin ulcers becomes difficult (Eming et al. 2014; Markeson et al. 2015; Límová 2010). Moreover, the skin barrier function and appearance of patients were seriously affected, which, keeps the body from the state of self-stabilization, and even finally lead to death (Eming et al. 2017). Therefore, it makes a great difference to cover the wound and restore skin barrier function. At present, skin wound repair remains one of the most difficult problems that needs to be addressed in the field of tissue repair (DiPietro 2016; Karin and Clevers 2016). Autologous skin transplantation is a common method for wound repair, but it cannot solve the scar hyperplasia caused by dermal tissue loss (Kohlhauser et al. 2021; Rippa et al. 2019).

Transplantation of dermal substitute is an appropriate solution to dermal tissue loss and an important part of deep burn and severe trauma wound repair (Snyder et al. 2020; Sando and Chung 2017). Dermal substitute combined with blade-thickness skin transplantation is an effective way to reduce scar hyperplasia and improve the quality of wound repair (Li et al. 2021). The dermal substitutes are mainly divided into natural biomaterials, biosynthetic materials and artificial synthetic materials, based on their sources. Each type of dermal substitute has its own advantages and disadvantages (Snyder et al. 2020; van der Veen et al. 2011; Nyame et al. 2015). Allogenic acellular dermis has proved to be a relatively ideal substitute for autologous dermis, application of allogeneic acellular dermis is restricted by its limited source (Huang et al. 2004; Kim et al. 2019; Lucke et al. 2015). Biosynthesis materials are usually made from proteins extracted from animal tissues, such as cowhide and pig skin. However, animal-derived materials carry the risks of hypersensitivity and pathogenic bacteria contamination (Morris et al. 2017; Rahmani Del Bakhshayesh et al. 2018). Artificial synthetic materials are generally inferior to natural materials in terms of biocompatibility and cellular affinity. Degradation products of artificial synthetic materials may be cytotoxic and prone to inflammation and secondary local infection on the wound surface (Vig et al. 2017; Mogoşanu and Grumezescu 2014). Besides, all types of dermal substitutes share common disadvantages, including poor nutrient permeability, slow rate of vascularization, and low transplant survival rate (Frueh et al. 2018; Urciuolo et al. 2019).

Desirable dermal substitutes are generally characterized by a good biocompatibility, wide material sources, convenience for mass production, and rapid vascularization (Goodarzi et al. 2018). Moreover, dermal substitutes can provide an appropriate structure that could be repopulated by the body’s cells (Rippa et al. 2019; Nyame et al. 2015; Maisel Lotan et al. 2019). Among the dermal substitutes, natural biomaterials have natural advantages over biosynthetic materials and artificial synthetic materials in the prevention and treatment of local infection and cytotoxicity (Vig et al. 2017; Sharma et al. 2019; Debels et al. 2015). While animal-derived xenograft dermal matrix has a wide range of sources, its noticeable disadvantages are immune rejection and vascularization (Salvatori et al. 2015). Therefore, overcoming the above weaknesses is the key to ensure the supply of good dermal substitutes. Overall, developing a suitable dermal substitute has been not only a burning issue in clinical medicine, but also one of the focuses and hot spots in the field of the current medical and biomedical engineering (Su et al. 2019; Xu et al. 2019).

Decellularization is the most common approach to address the xenodermal immunogenicity, acellular dermal matrix is often prepared in combination with various methods. In order to obtain better cell removal rate and better stability. Among them, trypsin and neutral protease has attracted wide attention (Klama-Baryla et al. 2020).

The lack of effective vascularization pathway has always been considered as the main reason for the difficulty in vascularization of dermal substitutes, and drilling is considered an excellent solution to this problem (Shahin et al. 2020). However, large pore size will affect the spatial structure stability of dermal substitutes, leading to obvious scar after wound repair, and losing the original purpose of using dermal substitute (van der Veen et al. 2010). Therefore, micropores are considered to be an ideal solution to this problem. Studies have shown that the optimal size of ADM is generally between 100 and 200 μm (Han et al. 2014; Boekema et al. 2014; Bonvallet et al. 2014; Hilmi et al. 2013), Other studies have shown that less than 350 μm is a suitable choice (Hilmi et al. 2013). In short, micropores are universally recognized and laser is the ideal method to achieve this goal.

In recent years, porcine acellular dermal matrix (PADM) has been considered as a promising dermal substitute because of its availability and good biocompatibility. The present study prepared porcine acellular dermis by enzymatic hydrolysis and new matrix laser microporous treatment. We succeeded in overcoming the immune rejection and difficulties in vascularization of PADM scaffold, providing a new method to solve the problem of the deficiency of ideal wound cover sources.

## MATERIALS AND METHODS

### Preparation of porcine acellular dermal matrix (PADM)

An acellular dermal matrix was prepared from the skin under a sterile condition as described previously. Briefly, a healthy pig weighing 25 kg (provided by the Experimental Animal Center of Wenzhou Medical University) was sacrificed, and the 0.2 mm blade-thickness epidermis was then removed by using an electric dermatome (Zimmer, USA). Thereafter, the 0.3 mm thick dermis tissue was excised and used for the subsequent experiments.

To prepare the PADM, the 0.3-mm thick porcine dermis was treated respectively with trypsin solution, neutral protease solution and ionic detergent SDS solution to remove the cellular components in the dermis. After the treatment, only the dermal matrix with normal structure of collagen was retained. The preparation consisted of the following steps: (a) Enzymatic hydrolysis treatment: the porcine dermis was hydrolyzed with 0.25% trypsin at 37 °C for 4 h and then subjected to enzymatic hydrolysis with 2.5 U/mL Dispase at room temperature for 4 h; (b) Elution treatment: each elution treatment should be washed 7 times as follows, it was soaked in purified water and cleaned once for 5 min, followed by ultrasonic cleaning twice for 5 min each time, washing in purified water shaker twice for 5 min each time, and washing in the saline shaker twice for 5 min each time; and (c) Contamination treatment: it was soaked in 2% ionic stain remover SDS solution, kept in a 37 °C thermostatic oscillator for 12 h, and then removed. The above cycle was repeated twice during the preparation. Among all the steps involving enzymatic hydrolysis, elution, decontamination and elution, elution treatment is a cohesive process for the preparation.

The laser micropore porcine acellular dermal matrix (LPADM) (pore size, 135 μm; pore space, 1 mm) was prepared using a laser punch on the PADM, followed by sterilization using Co60 100 Gy dose irradiation. Both PADM and LPADM were histologically examined under the scanning electron microscope and stored at 4 °C for further treatment. All experimental procedures were compliant with the Guidelines on the Use of Live Animals in Research issued by the Institutional Animal Care and Use Committee (IACUC) of Wenzhou Medical University, as well as the *Guide for the Care and Use of Laboratory Animals* published by National Institutes of Health.

### Cell separation and culture

A healthy rat weighing 250 g (provided by the Experimental Animal Center of Wenzhou Medical University) was sacrificed by cervical vertebra dislocation. The dorsal skin of the rat was shaved, and normal skin specimens were excised and rinsed with sterile saline. Most of the subcutaneous fat and fascia tissue were removed with either a scalpel or eye scissors. For the separation of keratinocytes, the skin was cut into strips of 0.3 cm × 1 cm wide, placed in a petri dish with the epidermis side up, soaked in 0.25% trypsin and then incubated at 37 °C for 1 h (to the extent that the ophthalmic tweezers can gently peel off the epidermis). The digested specimens were placed on the ultra-clean worktable. After digestion, the trypsin was removed, and the specimens were rinsed twice with DMEM. Thereafter, the dermis was cut into very small pieces (1 mm^3^) with eye scissors, transferred into a collagenase Type II (200 IU/ml) solution and incubated at 37 °C for 2 h in an incubator until the tissue blocks were basically digested and dispersed. The D-hank balanced salt solution was added to the treated dermis, and the dermis was then centrifuged at 500 rpm for 5 min. The centrifugation was repeated 5 times to wash away the collagenase. In this case, precipitation is fibroblasts. The supernatant was removed, and the cells were cultured in DMEM-high glucose (4.5 g/L, Gibco, Grand Island, NY, United States) supplemented with 10% fetal bovine serum (FBS) and 1% penicillin–streptomycin (Solarbio life Science, Beijing, China). The isolated cells were counted on a hemocytometer and then seeded into culture dishes of high-sugar DMEM (4.5 g/L) at a density of 5 × 10^4^ cells/cm^2^. The cells were maintained at 37 °C in a humidified incubator containing 5% CO_2_, and the culture media were replaced three times per week during the culture period. For conventional passage, the cells were treated with Trypsin–EDTA for 5 min and then split into new dishes at a 1:3 dilution.

### DNA residue determination of PADM

The test of DNA residue determination met with the national standard of YY/T 0606.25–2014.

Preparation of reaction system and digestion of protease K. Preparation of recovery samples, the λ-DNA standard was gradient diluted with 1 × TE solution to prepare 8, 4, 2, 1, 0.5 and 0 ng/μl standard solution, respectively. Add 100 μl of Standard Solution and 40 μl of 3%BSA Solution to Protease K Digestive Solution 70 μl, and vortex blend well. Preparation of samples to be tested, PADM was cut into two copies of 1 cm × 1 cm, cut into pieces and put into 1.5 ml sterile centrifuge tube. Among them, one was used for DNA extraction and the other was dried to a constant weight in an oven at 37 °C, and the dry weight was measured. Add 100 μl of the PADM debris and 40 μl DEPC water to Protease K Digestive Solution 70 μl and vortex blend well. Proteinase K digestion, the above two reaction systems were digested in a water bath at 56 °C until the samples to be tested were completely digested and no particles were visible to the naked eye(digestion time about 65 min).

### Purification of DNA

DNA purification was performed using the PrepSeqTM accounting extraction kit, and the specific operation was carried out in accordance with the instructions of the kit. When the DNA was finally dissolved, 150 μl of the dissolved solution was added to the sample, followed by eddy vibration for 5 min and water bath at 70 °C for 7 min. Centrifuge at high speed for 2 min, place on a magnetic frame for 2 min, and transfer the supernatant to a new set of centrifuge tubes. The supernatant is the extracted DNA (Storage at -20 °C).

### Determination of DNA content (fluorescence staining)

Preparation of DNA standard curve sample and standard solution, mix λ-DNA standard into 0, 50, 100, 200, 400, 800 ng/μl standard solution with 1 × TE solution, add 125 μl of the diluted standard solution to 96-well plate, each sample has 3 replicates. To ensure that the determination range of recovery group was within the standard curve, the samples of recovery group were diluted 20 times, then add the diluted recovery group solution and PADM group solution 125 μl per well to a 96-well plate, each sample has 3 replicates. Add Reagent fluorescence staining solution 125 μl to the 96-well plate respectively (mixed with sample in equal volume), shock and mix, and then incubate for 5 min in dark at room temperature, and determine with fluoresce plate. The test conditions are: Taking 480 nm as the excitation wavelength, 520 nm as the emission wavelength and 530 nm as the cut-off wavelength, the measurement were carried out, and the obtained data were graphed and analyzed. The fluorescence intensity measured with 1 × TE solution as the background value was used to measure and record the relative fluorescence intensity (RFI) of each measured hole.

### Cytotoxicity test

Cytotoxicity test met with the national standard of GB/T 16,886.5–2017.

A 100 μl cell suspension at a density of 5 × 10^4^ cells/mL was added to each well of a 96-well plate (5000 cells/well). The cells were seeded in the plate under continuously shaking.

The LPADM was cut into flakes (2.0 cm × 3.0 cm × 0.3 mm) and washed with PBS twice for 15 min each time in the generator. The flakes were placed into sterilized 5 ml centrifuge tubes, and 2 ml of complete medium was added to each centrifuge tube. Thereafter, these tubes were placed in a 37 °C shaker at 100 rpm, and the flakes were soaked and extracted for 24 h. All the tubes were divided into five groups: Negative tube: 2 mL complete medium + EP tube; Positive tube: 1.8 ml complete medium + 200μL DMSO (10%); Blank tube: 2 mL complete medium only; LPADM tube: 2 mL complete medium + 2 cm × 3 cm LPADM; PADM tube: 2 mL complete medium + 2 cm × 3 cm PADM. After 24 h of extraction, the culture medium was completely sucked out and 100 μl of the LPADM extraction was added to each well. The sample wells and control wells were both set with 6 replicates, and incubated in an incubator at 37 °C with 5% CO_2_ for 24 h, 48 h and 72 h, respectively. After each incubation, the extraction was sucked out, and 110 μl of CCK-8 reagent (1 mL CCK-8 + 10 mL medium) preheated in a 37 °C water bath was added to each well. The mixture was then incubated in a 5% CO2 incubator at 37 °C for 2–4 h, and the OD450 nm value was measured by an enzyme-labelling measuring instrument.

### Immunohistochemical staining

After deparaffinization and rehydration, skin tissue sections were submerged in 3% hydrogen peroxide at room temperature for 15 min to inactivate the endogenous peroxidase of the sections, and then soaked in 5% BSA to block nonspecific binding sites at 37 °C for 30 min. Subsequently, sections were incubated at 4 °C overnight with antibody against CD31 (1:100), and TNF-α (1:300). The sections were then rinsed with PBS, and incubated at 37 °C for 60 min with biotinylated secondary antibodies that diluted with PBS (1:1000), corresponding to the first antibodies, respectively. Finally, the reaction was stopped by a DAB Chromogen Kit for all sections for 8 s to 3 min, and the tissue sections were counterstained with hematoxylin and mounted with neutral resin. Images were captured by a Nikon light microscope (ECLPSE 80i, Nikon, Japan), and analyzed by an Image-Pro Plus software (Nikon, Tokyo, Japan).

### Transplantation of LPADM and PADM in the animal model

After general anesthesia with an intraperitoneal injection of 3% pentobarbitalnatrium (50 mg/kg body weight), the dorsal fur of SD rats weighing 200 to 250 g was shaved and split. The whole skin of the back was removed to the deep fascia to form a 2 cm × 2 cm wound. The subcutaneous fascia and part of dermis were removed to form a blade-thickness skin, and the skin was soaked in normal saline for later use. Blade-thickness skin and PADM/LPADM were transplanted into the wound. Thereafter, the wound was covered with vaseline gauze and chloramphenicol gauze, packaged and fixed. All the rats were raised in a single cage. The rats were divided into two groups with 12 rats for each group: Experimental group and Control group. For the Experimental group, one-step method was used to complete the transplantation of rat LPADM and autologous blade-thickness skin. Meanwhile, one-step method was used to complete the transplantation of PADM and autologous blade-thickness skin in the Control group. The samples were collected 14 days after transplantation.

### Statistical analysis

All data were expressed as mean ± standard error of the mean (SEM). Statistical differences were performed using one-way analysis of variance (ANOVA) followed by Tukey's test with GraphPad Prism 5 software (GraphPad Software Inc., USA). For all tests, *p value < 0.05, **p value < 0.01, ***p value < 0.001.

## Results

### Gross and morphological observation of PADM

**Fig. 1:**
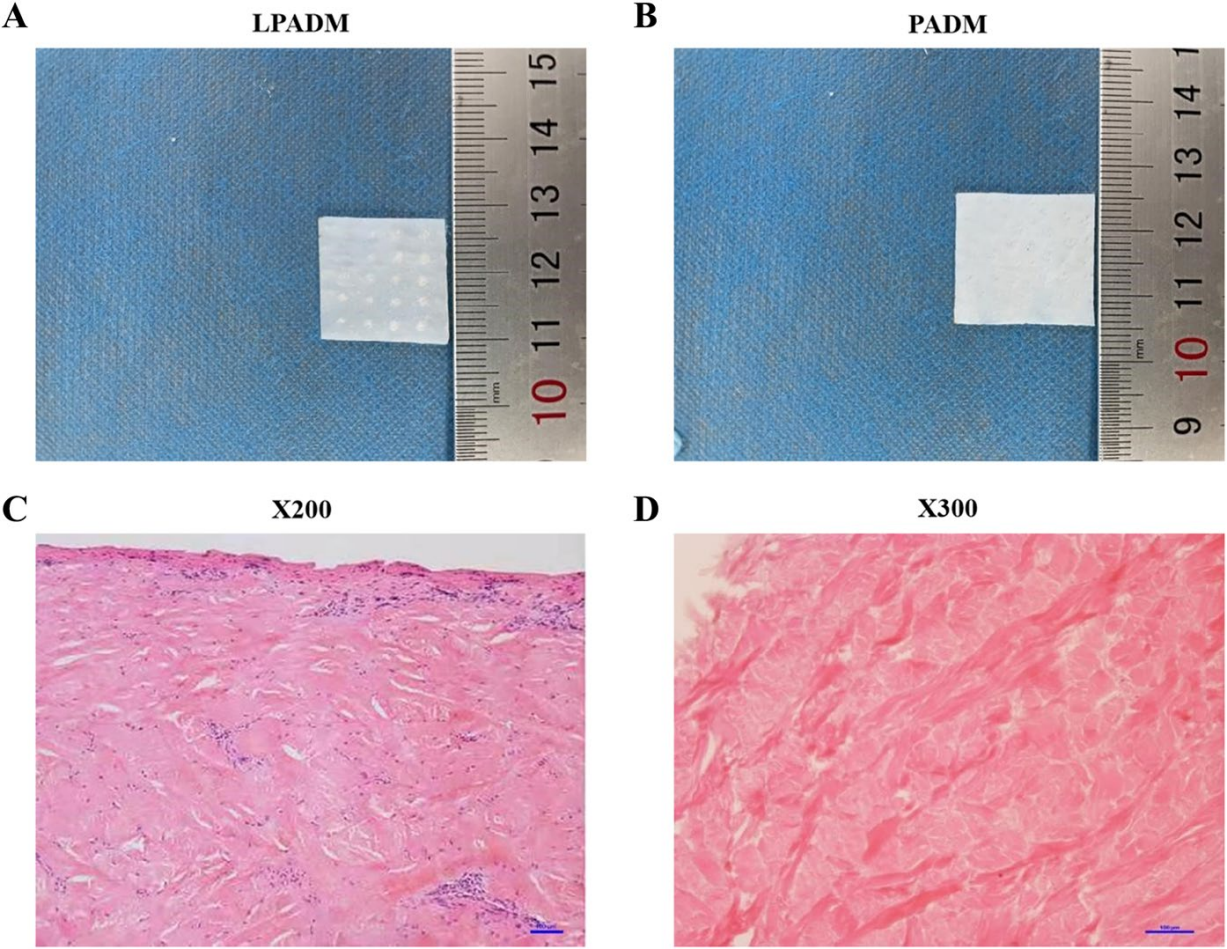
Gross and morphological observation of porcine dermis and acellular dermal matrix (ADM). Hematoxylin and eosin staining showed the morphological feature of porcine dermis and acellular dermal matrix under × 200 and × 300 magnification

A porcelain white, shiny, soft and elastic PADM was prepared in this study. HE staining and SEM observation were performed to characterize the acellular property of PADM. As shown in Fig. 1C, the cell structure of Porcine dermal tissue was intact, while the blue nucleus and light red collagen structure were tightly arranged. Moreover, the collagen bundles were intact and well organized. On the contrary, no cells were observed in the PADM tissue and only cell debris was retained (Figs. 1D).

**Fig. 2:**
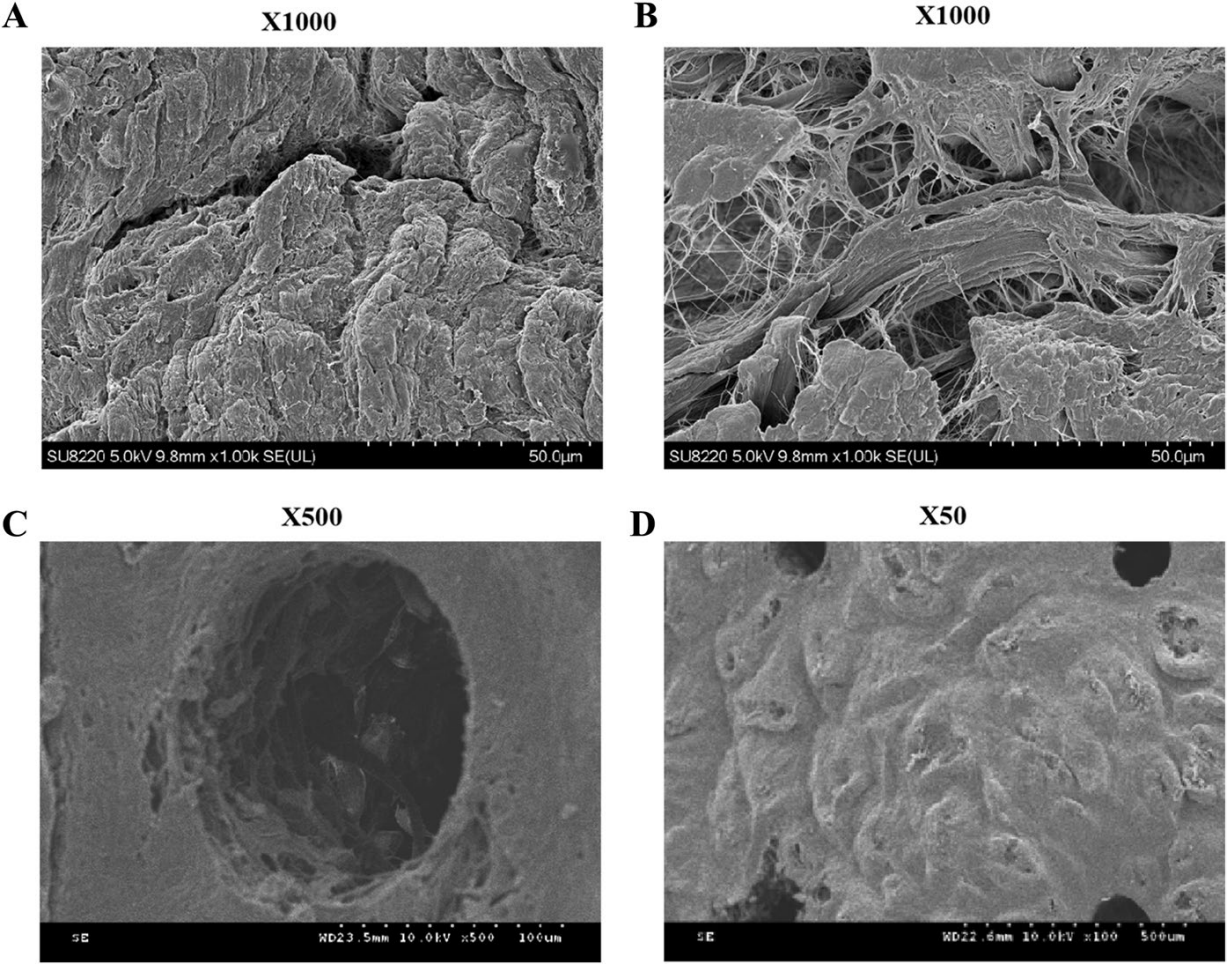
Morphological observation of porcine dermal matrix (A), porcine acellular dermal matrix (PADM, B) and laser micropore porcine acellular dermal matrix (LPADM, C and D). Scanning electron microscopy revealed the structural characterization of PADM and LPADM under × 100, × 500 and × 1000 magnification

The SEM observation showed that the Porcine dermal tissue had irregular surface and a cord-like structure of the collagen fibers (Fig. 2A), while the PADM displayed orderly arranged collagen with no obvious fracture (Fig. 2B).

PADM was interwoven with fascicular-like structures to form a grid-like spatial framework. This phenomenon showed that the cell components of PADM were basically removed, while the collagen fibers were normally arranged, and the structure was intact. In the meantime, LPADM exhibited no obvious shrinkage after laser punch, and there was light carbonization degree of dermal tissue at the edge of micropores (less than 0.25 um) (Fig. 2C). The micropores were regularly round, uniform in size and arranged in an ordered matrix (Fig. 2D).

### DNA residue determination of PADM

Standard and recovery samples and PADM group were determined to obtain the relative fluorescence intensity value and take the average value. The fluorescence intensity value of the standard substance was subtracted from the background value, which was taken as the vertical axis (Y), and the addition amount (ng/ml) was taken as the abaxial axis (X) to draw the standard curve, Y = 65.06x + 299.5, R2 = 0.998.The recovery curve, y = 0.914X-17.21, R2 = 0.999, showed good linear relationship within the test range. The DNA content (ng) of the sample after purification was substituted into the recovery curve equation to obtain the DNA residue (ng) before purification.DNA residue (NG) before purification was used to obtain DNA residue per unit weight of the tested products (Table 1).

**Table 1:** DNA residual weight of each PADM sample

Sample	1	2	3	4	5	6	Average
DNA residues(ng/mg)	6.22	5.66	2.70	2.93	3.91	8.14	4.93

### Cytotoxicity test of LPADM

**Fig. 3:**
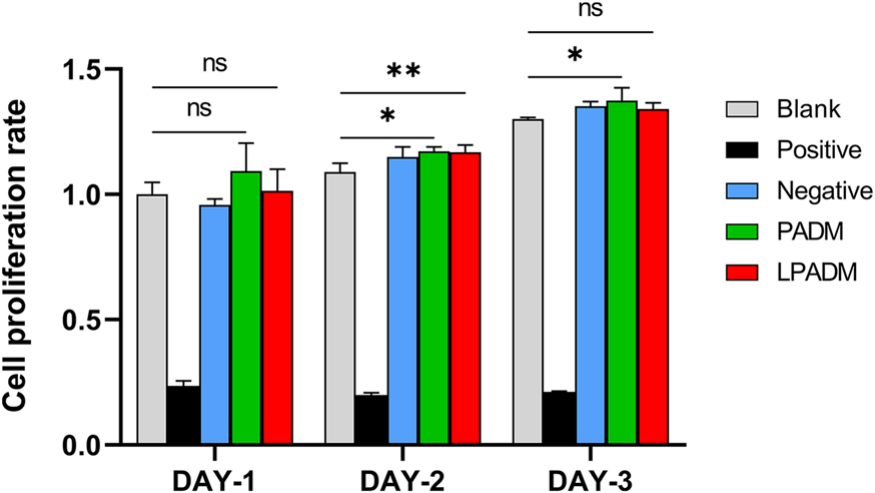
Comparison of the histograms of fibroblast growth in each group at 24, 48 and 72 h. * P < 0.05, ** P < 0.01

As summarized in Fig. 3, the cytotoxicity test revealed that after 24 h, 48 h, and 72 h of culture, the proliferation of fibrocytes in PADM and LPADM groups was observed, which showed no statistical difference compared with negative group. These data indicated that PADM and LPADM were almost no cytotoxicity.

### Characterization of the PADM/LPADM transplantation

**Fig. 4:**
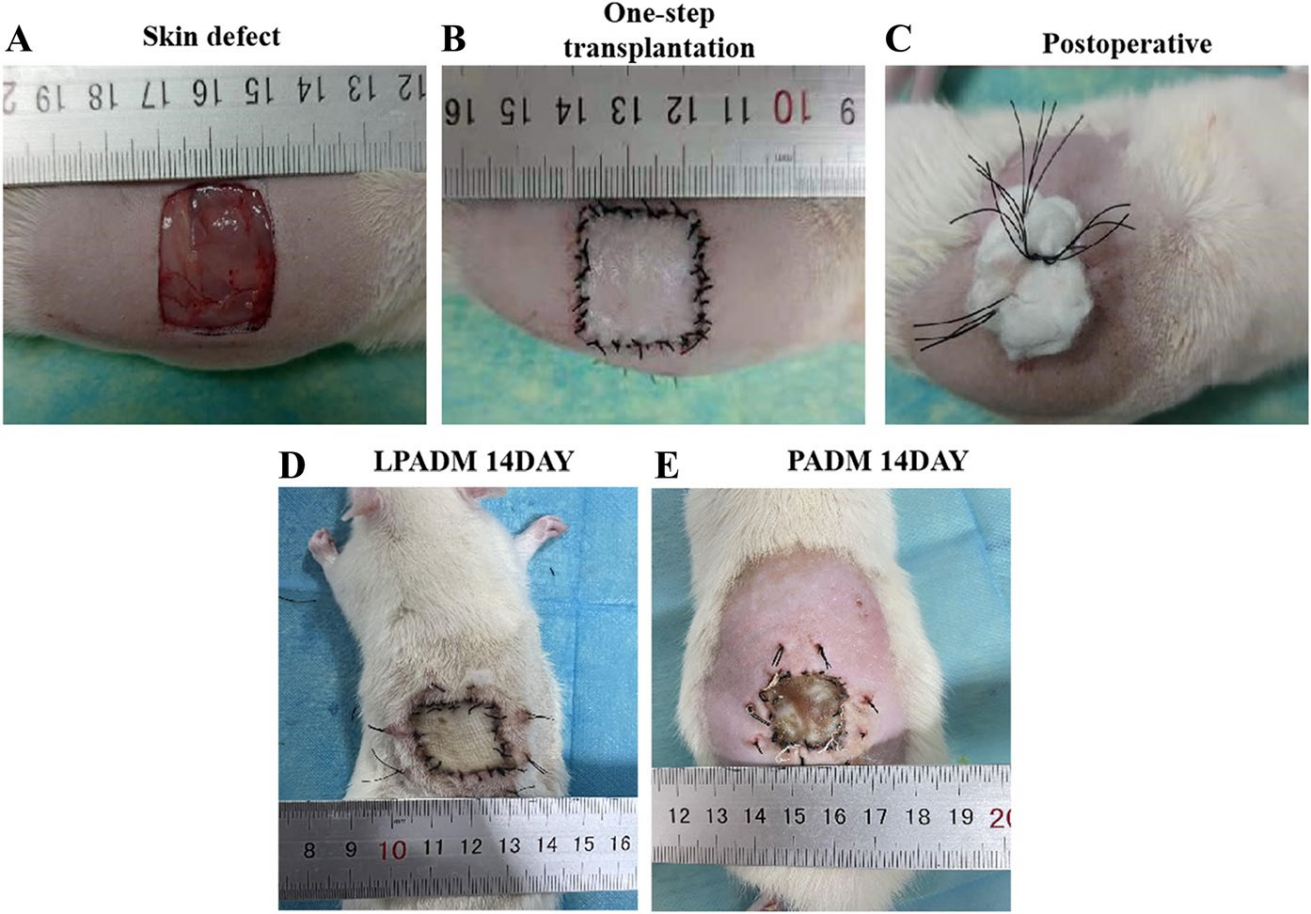
Animal model production process: macro images of (A) skin defect production, (B-C) one-step transplantion of dermal matrix with autogenous skin, (D) Postoperative. General view of blade-thickness skin at 14 days after transplantation in LPADM group and PADM group (E–F)

**Fig. 5:**
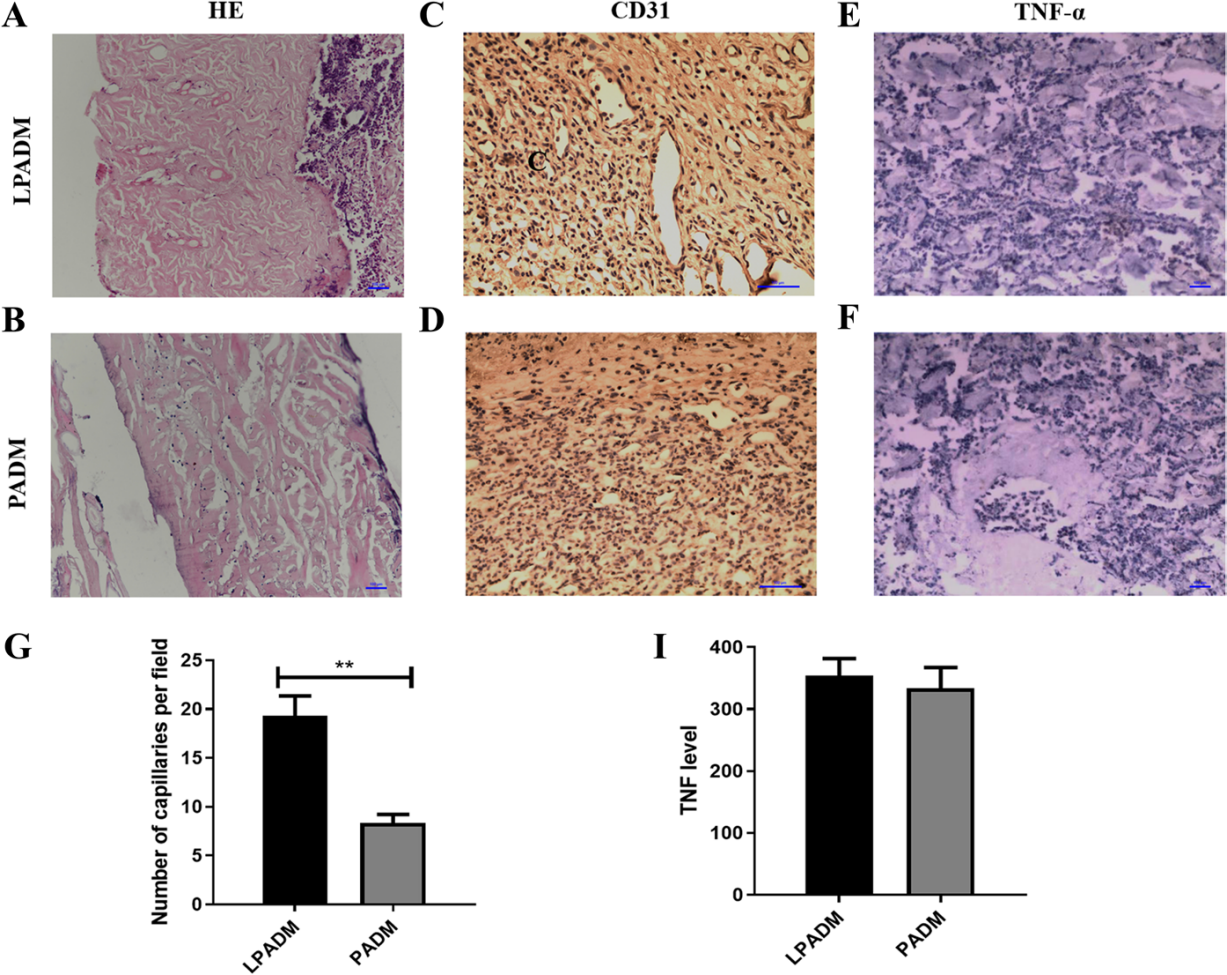
(A-B) Hematoxylin and eosin staining for the two groups on day 14 (Scale bar = 100 µm). (C-D) Immunohistochemical staining of CD31 for the two groups on day 14 (Scale bar = 100 µm). (E–F) Immunohistochemical staining of TNF-α for the two groups on day 14 (Scale bar = 100 µm). (G-H) Quantitative results of CD31 and TNF-α for each group. ** P < 0.01

As shown in Fig. 4, LPADM grafts in the experimental group were well healed and vascularized 14 days after transplantation, while all the autologous skin grafts survived (Fig. 4D). Moreover, the skin in the operating area could be pulled and elastic after 1 month. In the meantime, PADM in the control group was white, and the skin graft was partially necrotic (Fig. 4E). Histological examination(Hematoxylin and eosin staining, CD31 Immunohistochemical staining) revealed that while LPADM in the experiment group was fully vascularized 14 days after transplantation(Fig. 5A, 5C), a few blood vessels in the dermal scaffold can be observed in the control group (Fig. 5B, 5D). The quantitative results showed that the number of capillaries in the LPADM group (27.3 ± 5.7) was statistically much higher than in the PADM group (14.3 ± 4.7) (Fig. 5G). No obvious absorption, degradation or encapsulation of PADM was found in each phase. The animals exhibited no abnormal daily activities and gained weight. No obvious local or systemic allergic reactions were observed in all experimental animals. Immunohistochemistry of TNF-α was negative in both groups, and there was no statistical difference between the two groups (Fig. 5H), indicating that the local inflammatory reaction was slight, which confirmed its satisfying biocompatibility (Fig. 5E, 5F).

## Discussions

At present, skin trauma such as severe trauma, extensive burns, and wound healing after orthopedics and diabetes remains a growing worldwide health problem that concerns the physical and mental health of patients as well as the productivity of society as a whole (Cheng and Fu 2018; Haalboom 2018; Bhardwaj et al. 2017). The above situation not only causes great harm to individuals, but also huge loss of life and property to the families, society and country. The therapeutic effect is directly related to the patient's future quality of life. Dermal substitute is very important for solving scars caused by burn and trauma (Rippa et al. 2019), although several autologous and allogeneic dermal substitutes have been used for skin reconstruction, dermal substitutet remains challenging due to the high incidence of complications with low graft survival (Gaudio et al. 2014). These difficulties are mainly due to the immunogenicity of the materials and the failure of rapid vascularization, resulting in autologous skin complex graft necrosis, which has become an urgent problem to be solved.

A satisfactory dermal matrix should satisfy two conditions: very low immunogenicity and high speed of effective vascularization (Cui et al. 2019). Due to the presence of cell-surface specific antigens, when the foreign tissue is implanted into the body as an "alien component", the graft will be recognized by the recipient's immune system, and will produce a series of attacks, destruction and elimination effects on it. The most important principle in the application of acellular xenodermis is its safety, and thorough decellularization is the basis for its safety. Incomplete acellular effect will lead to a series of immune rejection reactions after dermal substitute transplantation, and ultimately lead to the failure of transplantation. There are many kinds of decellularization technologies, but they are mainly divided into three categories: physical methods (mechanical stirring method, pressure method, supercritical fluid method, etc.); Chemical methods (non-ionic detergent, ionic detergent, zwitterionic detergent, acid, alkali, hypertonic brine, etc.); Biological methods (enzymes, chelating agents, toxins, etc.). These methods can be used alone or in combination, although combination results are better. The present study adopted sequential combined decellularization of trypsin, neutral protease and SDS solution method for decellularization and then fully rinsed by ultrasonic wave. DNA residue assay showed little DNA residue and low immunogenicity, and cytotoxicity test showed almost no cytotoxicity, which were superior to the national quality standard. The paraffin section revealed no epithelial or endothelial cell residues as well as good structural integrity and low immunogenicity. Meanwhile, the electron microscopy showed that the collagen fibers were orderly arranged and displayed a good structural integrity. Moreover, we did not observe death or infection, skin allergic reaction, and weight loss in the animal experiments. All these indicated that it had a good acellular effect and no obvious damage to the spatial arrangement of collagen.

It has been demonstrated that following implantation of skin or AMD into the wound, wound exudation, vascular network anastomosis and neovascularization provided the nutrient sources sequentially (Rowan et al. 2015; Yin et al. 2018; Swaim 1990). As an acellular product of the dermis of the fault, ADM has its own residual pore after hair, sweat glands, sebaceous glands and microvessels were removed. Given that the pore is few and does not directly penetrate the whole dermal matrix, it is difficult for the basal plasma and other exudates of the wound to pass through ADM quickly after implantation. As a result, the nutritional needs of the transplanted skin or epidermal cells at the early stage (1–2 days) cannot be effectively met. In contrast to skin grafts, the vascularization of dermal substitutes is slow and dependent on the ingrowth of vessel-forming angiogenic cells (Frueh et al. 2018). Collectively, these factors may lead to inadequate nutrient supply, slow proliferation and even death and shedding of some transplanted autologous skin or epidermal cells. Therefore, improving the permeability and vascularization rate of ADM is the key to increase the survival rate of the transplants. For this purpose, some clinical doctors used ADM pulling mesh and then overlapped autologous mesh transplantation, and appropriately pressurized dressing. In this case, the autologous skin was in contact with the base of the wound via the ADM mesh to complete the absorption of nutrients. Due to the large size of the mesh, the wound healing can still form netlike or punctual paralysis scars, thus reducing the application value of ADM as a dermal scaffold.

Obviously, smaller pores are more conducive to reducing scarring, but too small pores can affect the vascularization of dermal stents. Therefore, the appropriate pore size is vital to improve the vascularization rate of acellular dermal matrix (ADM) for preparing ADM. In recent years, with the development of laser, micropores have become a reality. In recent years, the study of appropriate pore size has gradually come into people's field of vision. Meanwhile, a large number of studies have been conducted both at home and abroad to investigate the influence of the pore size. A study examined the effect of the pore size on cell proliferation and differentiation using scaffolds with various pore sizes. Notably, researchers have found that scaffolds with small pore size were beneficial to cell migration and the initial proliferation rate of cultured cells was increased (Mehr et al. 2015). The larger pore size facilitates rapid vascularization of dermal stents. However, larger is not always better. Particularly, the enlarged pore size not only leads to scar hyperplasia, but also compromise the physical properties of dermal scaffold. Wang et al. used type I collagen as raw material to prepare monolayer scaffolds with different pore sizes for both in vitro and in vivo experiments. While scaffolds with a diameter of 166.9 μm displayed the highest number of adherent and proliferating cells, those with a diameter of 100 μm showed good effect on wound healing when the pores were oriented perpendicular to the wound passage(Wang et al. 2015). Bonvallet et al. prepared the scaffold of collagen and electrospinning with an optimal pore size of 160 μm (Bonvallet et al. 2014). The optimal size of ADM is generally considered to be between 100 and 200 μm. In the present study, the size of LPADM was 135 μm, which was similar to the previous literatures.

In order to improve the vascularization rate of PADM, this study prepared the novel LPADM by using in a special laser drilling technology that has obtained a national utility model patent. Electron microscope results showed that LPADM exhibited no obvious shrinkage after laser punch, and there was light carbonization degree of dermal tissue at the edge of micropores. The micropores were regularly round, uniform in size and arranged in an ordered matrix, less destruction of tissue structure. Therefore, the experimental method preserved the structural integrity of the dermal scaffold to the maximum extent while acellular. In the in vivo transplant experiment, LPADM autologous skin transplanted by one-step method survived well of the experiment group, and the LPADM was fully vascularized. Notably, LPADM exhibited very good vascularization activity and high safety in animal transplantation experiments. Conversely, we found that the survival rate of PAMD grafts in the control group was low, Also, it is noteworthy that the vascular growth in the control group was significantly slower than that in the experiment group, albeit there was still some vascular growth in the grafts. The results revealed that the one-step method led to a higher survival rate, less damage of tissue structure and less scar than PADM.

There are other studies, in 2007, a research group performed a systematic study on the empty space of the dermal matrix with a thickness of 0.3 mm-0.4 mm. The study demonstrated that while the empty space of 0.8–1.0 mm could improve the quality of composite transplantation with blade-thickness skin, the empty space of 1.0 mm displayed the best effect (Liang et al. 2007).

Nevertheless, several limits of our study should be mentioned. First; we mainly consider the clinical practice, from the perspective of composite skin grafting, not only to individual decellularized dermal matrix set transplantation group compared. second, there is no systematic comparison of the effects of different pore sizes, and the data sources are more references. Finally, even though we have used the combination of maximum out of cells in the form of cell and heterologous DNA content is lower than the national standard. However, due to the presence of our allogenic DNA, this still affects the immunogenicity of allogenic acellular dermal matrix to some extent.

In conclusion, use of heterogeneous dermal tissues (e.g., pigs) as ADM preparation materials, thorough acellular reduction of ADM immunogenicity and drilling for improving the rate of vascularization should be considered as the preferred method in terms of ease of use. This method not only takes into account the disadvantages and advantages of pulling mesh, but also builds the rapid vascularization pathway system in the dermal tissue structure. The early use of nutrient solution and sufficient drainage reduces the accumulation of local exudate. In terms of reducing scarring and long-term recurrence, the preferred pore size is currently 135 μm, this parameter needs to be further determined in larger studies.

## Data Availability

All data generated or analyzed during this study are included in this published article.
